# The same or different psychiatrists for in- and out-patient treatment? A multi-country natural experiment

**DOI:** 10.1017/S2045796018000732

**Published:** 2018-12-18

**Authors:** D. Giacco, V.J. Bird, T. Ahmad, M. Bauer, A. Lasalvia, V. Lorant, E. Miglietta, J. Moskalewicz, P. Nicaise, A. Pfennig, M. Welbel, S. Priebe

**Affiliations:** 1 Unit for Social and Community Psychiatry (World Health Organisation Collaborating Centre for Mental Health Services Development), Queen Mary University of London, London, UK; 2 Newham Centre for Mental Health, London, E13 8SP, UK; 3 Queen Mary University of London, Pragmatic Clinical Trials Unit, London, UK; 4 Centre for Primary Care and Public Health, Queen Mary University, London (Whitechapel Campus), Yvonne Carter Building, 58 Turner Street, London E1 2AB, UK; 5 Department of Psychiatry and Psychotherapy, Carl Gustav Carus University Hospital, Technische Universität Dresden, Universitätsklinikum Carl Gustav Carus, Klinik und Poliklinik für Psychiatrie und Psychotherapie, Fetscherstraße 74, 01307 Dresden, Germany; 6 UOC Psichiatria, Azienda Ospedaliera Universitaria Integrata di Verona, Piazzale Aristide Stefani, 1, 37126 Verona VR, Italy; 7 Institute of Health and Society IRSS, Université Catholique de Louvain, “Ecole de Santé Publique”, Clos Chapelle-aux-champs, 30 bte 30.15 - 1200 Woluwe-Saint-Lambert, Brussels, Belgium; 8 Section of Psychiatry, Department of Public Health and Community Medicine, University of Verona, Verona, Italy; 9 Institute of Psychiatry and Neurology, ul. Sobieskiego 9, 02-957 Warsaw, Poland

**Keywords:** Health services, outcomes, personal continuity, specialisation, treatment

## Abstract

**Aims:**

A core question in the debate about how to organise mental healthcare is whether in- and out-patient treatment should be provided by the same (personal continuity) or different psychiatrists (specialisation). The controversial debate drives costly organisational changes in several European countries, which have gone in opposing directions. The existing evidence is based on small and low-quality studies which tend to favour whatever the new experimental organisation is.

We compared 1-year clinical outcomes of personal continuity and specialisation in routine care in a large scale study across five European countries.

**Methods:**

This is a 1-year prospective natural experiment conducted in Belgium, England, Germany, Italy and Poland. In all these countries, both personal continuity and specialisation exist in routine care. Eligible patients were admitted for psychiatric in-patient treatment (18 years of age), and clinically diagnosed with a psychotic, mood or anxiety/somatisation disorder.

Outcomes were assessed 1 year after the index admission. The primary outcome was re-hospitalisation and analysed for the full sample and subgroups defined by country, and different socio-demographic and clinical criteria. Secondary outcomes were total number of inpatient days, involuntary re-admissions, adverse events and patients’ social situation. Outcomes were compared through mixed regression models in intention-to-treat analyses. The study is registered (ISRCTN40256812).

**Results:**

We consecutively recruited 7302 patients; 6369 (87.2%) were followed-up. No statistically significant differences were found in re-hospitalisation, neither overall (adjusted percentages: 38.9% in personal continuity, 37.1% in specialisation; odds ratio = 1.08; confidence interval 0.94–1.25; *p* = 0.28) nor for any of the considered subgroups. There were no significant differences in any of the secondary outcomes.

**Conclusions:**

Whether the same or different psychiatrists provide in- and out-patient treatment appears to have no substantial impact on patient outcomes over a 1-year period. Initiatives to improve long-term outcomes of psychiatric patients may focus on aspects other than the organisation of personal continuity *v*. specialisation.

## Introduction

Following major reforms over the past 50 years, mental healthcare systems in many countries provide acute treatment in hospitals and varying types of longer-term out-patient care (Killaspy, 2006). A core question in the debate about how best to organise mental healthcare is whether in- and out-patient treatment should be provided by the same (personal continuity) or by different psychiatrists (specialisation). The debate is controversial and has driven organisational changes in several European countries (Khandaker *et al*., 2009; Schmidt-Kraepelin *et al*., 2009; Karow *et al*., 2012; Killaspy, 2012; Lodge, 2012; Begum *et al*., 2013). However, these changes have gone in opposite directions.

For instance, in Germany in- and out-patient treatments have traditionally been provided by different psychiatrists. Arguing that this leads to fragmented and ineffective care, there have been repeated initiatives to re-organise services and introduce new arrangements so that the same psychiatrist can be responsible for in- and out-patient treatment (Schmidt-Kraepelin *et al*., 2009; Karow *et al*., 2012).

In contrast, in the National Health Service (NHS) in the UK, traditionally the same consultant psychiatrist was responsible for in- and out-patient treatment of all patients in a given catchment area. Over the past 10 years the majority of NHS services have been re-organised and now have different psychiatrists for in- and out-patient treatment, partly with the intention to improve struggling in-patient services (Royal College of Psychiatrists and National Institute for Mental Health, 2005; Department of Health, 2007; Burns, 2010; Killaspy, 2012; Laugharne and Pant, 2012; Lodge, 2012; Rosen *et al*., 2013). In other countries similar discussions take place and influence re-organisations (Antunes and Moreira, 2011).

These re-organisations can absorb much time and energy and generate considerable costs. So far, the debate and actual re-organisations have been guided by very limited research evidence. A systematic review of studies comparing personal continuity and specialisation (Omer *et al*., 2015) suggested that patients and clinicians tend to prefer personal continuity. However, no definitive conclusions could be drawn on clinical outcomes. Studies had serious methodological shortcomings as they had been conducted with small samples and in specific local settings, with limited generalisability. Most importantly, all studies compared a newly implemented form of organisation (personal continuity or specialisation) with the given existing approach, and the outcomes were invariably more positive for the new experimental approach, irrespective of its type.

Randomised controlled trials would require changes to the existing routine practice for one of the two approaches and introduce ‘novelty bias’, potentially limiting the external validity of the findings. Thus, randomised controlled trials may not be the ideal design to test whether personal continuity or specialisation leads to more favourable outcomes in practice. Instead, a natural experiment can be used to assess outcomes of services that provide personal continuity or specialisation as established routine, rather than in experimental or novel programmes (Black, 1996; Victora *et al*., 2004; Bonell *et al*., 2011; Craig *et al*., 2012; Omer *et al*., 2015).

A natural experiment was conducted in five European countries: Belgium, England, Germany, Italy and Poland. We selected countries which differ in their healthcare systems, traditions, funding arrangements and policies so that the findings would not be dominated by one specific national context. In order to avoid confounding of the effects of the care organisation with national differences, both approaches are routinely provided in selected countries, so that the organisation of care varied within each of them.

This study aims to assess whether personal continuity or specialisation of psychiatrists is associated with more favourable patient outcomes over a 1-year period following an index hospitalisation. The primary outcome was re-hospitalisation. Secondary outcomes were: the total number of in-patient days, involuntary re-hospitalisation, adverse events and changes in patients’ social situation.

## Methods

### Study design

The study is a natural experiment (Giacco *et al*., 2015) in which the exposure of patients to either approach is outside the control of the investigators.

In England and Italy, the allocation of patients to personal continuity or specialisation of their psychiatrists is strictly determined by the locality of their treatment and is the same for all patients living in the same area of residence. In Belgium, Germany and Poland, the allocation can vary for different patients within the same hospital. It is based on a clinical decision or can be influenced by insurance arrangements, local practices and service arrangements or be a patient's choice. Patients entered the study following admission to a psychiatric in-patient ward, and were prospectively followed-up for 1 year.

Ethical approval was obtained in all countries: (1) Belgium: Comité d'Ethique hospitalo-facultaire des Cliniques St-Luc; (2) England: NRES Committee North East – Newcastle & North Tyneside (ref: 14/NE/1017); (3) Germany: Ethical Board, Technische Universität Dresden; (4) Italy: Comitati Etici per la sperimentazione clinica (CESC) delle provincie di Verona, Rovigo, Vicenza, Treviso, Padova and (5) Poland: Komisja Bioetyczna przy Instytucie Psychiatrii i Neurologii w Warszawie.

### Settings

Fifty-seven hospitals were included across the five countries. The geographical location of the hospitals and their care approaches are reported in the protocol paper (Giacco *et al*., 2015).

The hospitals included were identified through contacts of the national research groups. In England and Italy, the hospitals were purposively sampled based on care approach in order to have even numbers of hospitals using personal continuity and specialisation. In Belgium, Germany and Poland personal continuity and specialisation were used within the same hospital.

A list of hospitals with care approaches used is provided in the online Supplementary material.

### Participants

The inclusion criteria for patients were:

1. 18 years of age or older;
2. clinical diagnosis of psychotic disorder (F20–29), affective disorder (F30–39) or anxiety/somatisation disorder (F40–49) according to the International Classification of Diseases-ICD-10 (World health Organisation, 1992);
3. being hospitalised in a general adult psychiatric in-patient unit;
4. sufficient command of the language of the host country to provide written informed consent and understand the questions in the research interviews;
5. capacity to provide informed consent.

Exclusion criteria were:

1. diagnosis of organic brain disorders;
2. too severe cognitive impairment for providing information on the study instruments.

### Interventions to which patients were exposed

Personal continuity, i.e., a patient is under the care of the same psychiatrist for in- and out-patient treatment; or specialisation, i.e., a patient is under the care of different psychiatrists for in- and out-patient treatment.

### Outcomes

#### Primary outcome

The primary outcome was readmission to hospital (binary: yes/no) within 1 year following the index admission.

#### Secondary outcomes

Secondary outcomes measured over the 1-year period were:

1. total number of in-patient days;
2. involuntary readmission measured using an *ad hoc* schedule based on the Client Service Receipt Inventory (Chisholm *et al*., 2000);
3. adverse events, measured as any adverse event such as death, completed suicide, suicide attempt or serious side effects of treatment requiring somatic or psychiatric hospitalisation;
4. the patients’ social situation, using the SIX index (Priebe *et al*., 2008) which varies from 0 (very poor social situation) to 6 (very good social situation) and captures: (a) employment (none; voluntary or protected or sheltered work; regular employment); (b) accommodation (homeless or 24 h supervised; sheltered or supported accommodation; independent accommodation); (c) living situation (living alone; living with a partner or family); (d) contacts with friends (not having met a friend within the past week; having met at least one friend in the past week).

### Procedures

#### Establishing exposure to personal continuity or specialisation of psychiatrist

In England and Italy, the allocation was determined by the locality of the hospital.

In Belgium, Germany and Poland, allocation was obtained from the primary hospital clinician report at discharge.

#### Recruitment and data collection

Patients consecutively admitted to the participating hospitals from 1 October 2014 to 31 December 2015 were screened within 2 days of admission and recruited to the study. Patients were followed-up for 1 year following their index admission. The follow-up was completed by end of February 2017.

First contact between the patient and the research staff took place within 2 working days after the hospital admission to minimise potential selection bias due to early discharge. The initial contact could be postponed within the index hospital stay if patients wished so or were deemed by the clinician in charge to be too unwell to be contacted by a researcher.

**Fig. 1.**
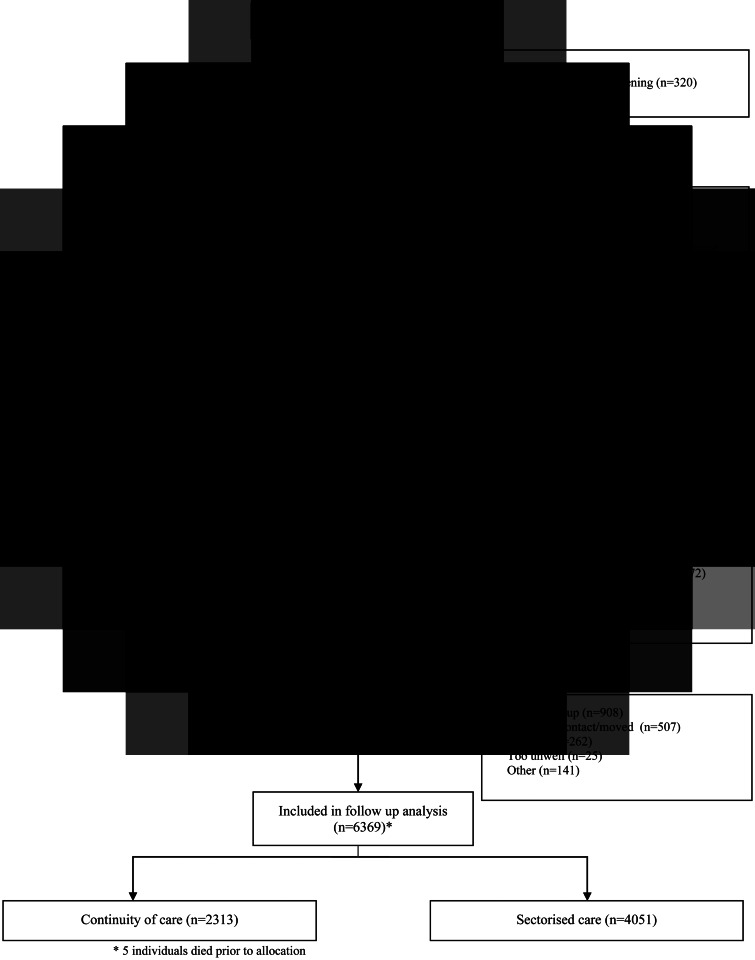
CONSORT diagram.

Before being contacted by a researcher, patients were asked by a clinician for their assent to participate in research. If assent was obtained, researchers were introduced to the patients, explained the study and obtained written informed consent for participation in face-to-face meetings. The CONSORT diagram with data on overall recruitment and data collection is reported in Fig. 1.

Follow-up data on the primary outcome were obtained from medical records in England and Italy and via phone or personal interviews in the other countries. For secondary outcomes medical records were primarily used in England with missing information being collected via phone or personal interviews. In the other countries secondary outcomes were collected via phone or personal interviews.

### Sample size determination

The sample size was calculated to detect a 5% difference in re-admission rates (i.e., proportion of patients re-hospitalised during the 1 year follow-up) with 80% power at the 5% significance level. The sample size required for an individual-level comparison not considering clustering of outcomes within centres and hospital would have been 2716 patients. Assuming a 15% drop-out rate and an inter-cluster correlation coefficient of 0.007 – resulting in a variance inflation factor of 1.90 (Adams *et al*., 2004) – the adjusted sample size was 5162 (1034 per country), requiring an average number of 150 patients recruited in eight hospitals per country, i.e., 1200 patients per country and 6000 overall.

Given the complex organisation and uncertain follow-up rates in this large study, we organised recruitment so that we ended with a higher number of recruited patients than envisaged in this calculation.

### Statistical analysis

The statistical analysis plan was signed off before the end of data collection (Giacco *et al*., 2015). The analysis was carried out using Stata version 15 (StataCorp, 2017). All inferential analyses were conducted two sided and significance interpreted at the 5% level. A random effect was incorporated into all analyses to account for the clustering of patients within hospitals.

The following characteristics were summarised for the whole sample and for each study arm: age, gender, marital status, highest completed education level, country of birth, employment, accommodation, living situation, friendships, self-reported benefits receipt due to low income, main psychiatric diagnosis at admission, severity of illness (Clinical Global Impression score at baseline) (Guy 2000), whether the index admission was the first admission and the formal status – i.e., voluntary or involuntary – at index admission. Baseline variables were compared between the two study arms, using analysis of variance for continuous variables and *χ*^2^ test for dichotomous variables.

Socio-demographic and clinical variables at baseline were compared between patients that withdrew from the follow-up and those that remained in the study. The number of and reasons for withdrawal were summarised across the treatment groups. If 80% of the items of a composite score (e.g., SIX score) were available, we used pro-rating first, and then used the mean of the other items to replace the missing item on that scale.

For the primary outcome, participants who died during the follow-up or were hospitalised for the full year were considered as re-hospitalised, in order to reflect a negative outcome of treatment. We used multiple imputation to account for missing data.

#### Primary outcome analysis

The primary analysis followed the intention-to-treat principles (http://www.consort-statement.org/consort-statement/further-explanations/box6_intention-to-treat-analysis/) with multiple imputation. The intention to treat sample is defined for this study as the intended arrangement of care for patients, i.e., personal continuity or specialisation of the treating psychiatrists.

The primary outcome was analysed using a mixed effect logistic regression model with a random effect for hospital. The analysis was adjusted for baseline variables that had been pre-specified in the protocol (Giacco *et al*., 2015): age, gender, diagnostic group (World Health Organisation, 1992), whether or not a patient has been previously admitted, severity of illness at baseline (Guy 2000), social situation (SIX score), formal status of the patient at baseline, length of stay in hospital and country.

Further participant characteristics which were measured at baseline were added to the model in case of significant differences between the two arms at baseline. Univariable associations of these variables with the primary outcome variable were tested. In the multivariable regression model only variables that showed a univariate association with the outcome variable of the analysis at a significance level of *p* < 0.10 were included.

Odds ratios (OR), confidence intervals (CIs) and *p*-values for each outcome of patients exposed to personal continuity compared with specialisation were reported. Adjusted values of outcome variables based on the multivariable mixed effect logistic regression model were calculated. The level of statistical significance was set at *p* < 0.05 for all the analyses.

#### Subgroup analyses

In these analyses the primary outcome was compared between personal continuity and specialisation approach in subgroups of participants using the same approach as the primary analysis but only analysing available cases.

Subgroups (Giacco *et al*., 2015) were formed by (a) country (Belgium, England, Germany, Italy, Poland); (b) diagnostic group (defined as F20–29, F30–39 and F40–49 based on the ICD-10 (World Health Organisation, 1992); (c) gender (female *v*. male); (d) age (≤40 years; >40 years); (e) socio-economic status (being on benefits *v*. not being on benefits); (f) migrant status (born in the country of residence *v*. not born in the same country) and (g) type of admission (first admission *v*. repeat admission).

An additional planned subgroup analysis of participants with comorbid physical health conditions was dropped because of concerns about consistency and quality of information on physical comorbidities found in psychiatric clinical records.

#### Secondary outcome analyses

The same analysis principles were followed as per the primary outcome analysis. A mixed effects model as appropriate for the type of variable was employed. Linear regression for continuous outcomes (SIX score, in-patient bed days in 1 year), and logistic regression for binary outcomes (percentage of involuntary readmission and any adverse event) were used. The only exception was that in the comparison of the total number of in-patient days during the 1-year period, the model was not adjusted for the length of index stay (as the index admission was part of the total number of in-patient days).

We did not conduct subgroup analyses for secondary outcomes to limit the risk of multiple testing.

## Results

### Sample characteristics

In total, 7302 participants were recruited into the study; 4726 were exposed to specialisation and 2566 to personal continuity arm. The characteristics of the total sample and of patients in the two arms are presented in Table 1.

**Table 1.** Baseline characteristics of participants in the total sample and within specialisation and personal continuity arms

Baseline characteristics	Main sample	Personal continuity	Specialisation
	(*n* = 7302)	(*n* = 2566)	(*n* = 4726)
Age, mean (s.d.)	42.4 (14.3)	42.5 (13.9)	42.4 (14.5)
Gender, male, *N* (%)	3811 (52.3)	1368 (53.5)	2439 (51.7)
Partnership status, married/co-habiting, *N* (%)	1838 (25.3)	685 (26.9)	1152 (24.5)
Born in the same country, yes, *N* (%)	6298 (86.5)	2171 (84.8)	4118 (87.4)
Education level			
Primary school, *N* (%)	1259 (17.3)	470 (18.5)	785 (16.7)
Secondary school, *N* (%)	2983 (41.1)	1082 (42.5)	1898 (40.3)
Further education, *N* (%)	2907 (40.0)	949 (37.3)	1955 (41.5)
Accommodation			
Homeless, *N* (%)	358 (4.9)	138 (5.4)	217 (4.6)
Living situation			
Living alone, *N* (%)	2648 (36.5)	896 (35.1)	1746 (37.2)
Paid employment, *N* (%)	1993 (27.4)	615 (25.3)	1377 (30.4)
Receiving benefits, yes, *N* (%)	3863 (53.4)	1484 (58.4)	2370 (50.6)
Diagnosis at admission			
Psychotic disorders, *N* (%)	2991 (41.0)	1128 (44.0)	1857 (39.3)
Mood disorders, *N* (%)	3598 (49.3)	1218 (47.5)	2375 (50.3)
Anxiety, dissociative, stress-related, and somatoform disorders, *N* (%)	1337 (18.3)	458 (17.9)	878 (18.6)
First admission, yes, *N* (%)	2435 (33.6)	824 (32.3)	1608 (34.3)
Voluntary admission, yes, *N* (%)	5667 (77.8)	1960 (76.5)	3699 (78.5)
Clinical Global Impression score, mean (s.d.)	4.3 (1.2)	4.3 (1.2)	4.3 (1.2)
Length of stay, mean (s.d.)	39.4 (49.7)	39.8 (49.6)	38.8 (48.7)
SIX score, mean (s.d.)	3.7 (1.4)	3.6 (1.4)	3.7 (1.4)

s.d. = standard deviation.

There were statistically significant differences between the two arms with regard to age, marital status, whether or not participants received benefits and prevalence of different diagnoses.

### Primary outcome: re-hospitalisation

In total, 6369 participants were followed-up for 1 year, 87.2% of those recruited. Of the participants, 2313 were in the personal continuity arm, 4051 were in the specialisation arm and five were not allocated. In total, 2259 participants (35.5%) were readmitted to an in-patient psychiatric unit at least once within 1 year, 33 (0.5%) were still in hospital after 1 year and 77 died (1.2%), 25 of them were readmitted before death. Among those exposed to personal continuity 854 out of 2313 followed-up participants (37.0%) were readmitted, ten (0.4%) were still in hospital after 1 year and 38 (1.6%) died. Among those exposed to specialisation, 1405 out of 4051 followed-up participants (34.7%) were re-admitted, 19 (0.5%) were still in hospital after 1 year and 39 (1.0%) died. Overall participants considered as re-hospitalised for the purpose of the analysis were 2340 (36.8%) in the total sample, 886 (38.3%) in the personal continuity arm and 1454 (35.9%) in the specialisation arm.

The adjusted percentages of re-hospitalisation were 38.9% in personal continuity and 37.1% in specialisation. The difference was not statistically significant (adjusted OR 1.08; CI 0.94–1.25; *p* = 0.28).

### Subgroup analyses

The socio-demographic and clinical characteristics for all considered subgroups are presented in Supplementary Tables 1–7. The re-hospitalisation rates in all subgroups are shown in Table 2.

**Table 2.** Primary outcome readmission^a^ by subgroups

	*N*	*N* readmitted (%)	Unadjusted proportion (*n*, %)		Adjusted proportion (%)		Result from regression model^b^
			Personal continuity	Specialisation	Personal continuity	Specialisation	Adjusted OR^a^ (95% CI), *p* value
Country							
Belgium^c^	726	317 (43.7)	159 (45.0)	154 (41.7)	45.8	44.1	1.13 (0.79–1.61), *p* = 0.52
England	2706	815 (30.1)	365 (33.4)	450 (27.9)	33.4	28.4	1.23 (0.96–1.58), *p* = 0.11
Germany	724	297 (41.0)	79 (47.9)	218 (39.0)	46.2	40.5	1.16 (0.77–1.74), *p* = 0.47
Italy	1108	488 (44.0)	173 (47.7)	315 (42.3)	47.5	42.3	1.10 (0.79–1.53), *p* = 0.58
Poland	1105	427 (38.7)	110 (32.5)	317 (41.4)	34.6	40.4	0.97 (0.66–1.43), *p* = 0.90
Diagnostic groups							
Psychotic disorders	2614	1040 (39.8)	420 (41.3)	617 (38.7)	42.4	40.5	1.07 (0.86–1.32), *p* = 0.55
Mood disorders	3147	1120 (35.6)	408 (37.2)	710 (34.7)	35.5	37.8	1.08 (0.88–1.32), *p* = 0.47
Anxiety, dissociative, stress-related, and somatoform disorders	1118	383 (34.3)	140 (34.3)	243 (34.2)	34.7	34.9	1.04 (0.77–1.42), *p* = 0.78
Gender							
Female	3026	1128 (37.3)	411 (38.6)	714 (36.5)	38.5	37.2	1.12 (0.91–1.38), *p* = 0.28
Male	3329	1210 (36.4)	473 (38.2)	736 (35.2)	39.2	37.0	1.03 (0.84–1.27), *p* = 0.78
Age							
≤40 years	2907	1074 (37.0)	399 (37.6)	672 (36.4)	39.4	37.8	1.01 (0.83–1.24), *p* = 0.90
>40 years	3462	1270 (36.7)	487 (38.9)	782 (35.4)	38.5	36.5	1.16 (0.95–1.42), *p* = 0.15
Socio-economic status							
Being on benefits	3398	1387 (40.8)	577 (42.8)	806 (39.4)	43.2	41.6	1.18 (0.98–1.43), *p* = 0.08
Not being on benefits	2918	944 (32.3)	304 (32.1)	640 (32.5)	32.8	33.2	0.97 (0.78–1.21), *p* = 0.80
Migrant status							
Migrant	881	288 (32.7)	127 (35.8)	161 (30.6)	36.4	33.0	1.29 (0.94–1.78), *p* = 0.12
Non-migrant	5471	2050 (37.5)	757 (38.8)	1289 (36.7)	39.3	37.7	1.08 (0.92–1.27), *p* = 0.34
Type of admission							
First admission	2106	526 (25.0)	190 (25.8)	335 (24.5)	26.0	25.2	1.04 (0.81–1.33), *p* = 0.77
Repeated admission	4221	1800 (42.6)	692 (44.2)	1105 (41.7)	44.8	43.3	1.11 (0.93–1.32), *p* = 0.25

a Patient who died during the study participation or were still in hospital 1 year from index admission were considered as re-hospitalised.

b The multivariable regression model was carried out on available cases and adjusted for the following factors: age, gender, diagnostic group, severity of illness at baseline, first/repeat admission, social situation (SIX score), formal status of patient at baseline and length of hospital stay), marital status, highest education level, migrant status and self-reported benefits receipt due to low income. The reference group was specialisation.

c *N* = 5 participants in Belgium were not allocated to either care approach at baseline.

No statistically significant differences were found in re-hospitalisation between personal continuity and specialisation within each country, within diagnostic subgroups, in female and male participants, in participants who were 40 years old or younger, in those who were older than 40 years, in those who were on benefits and in those who were not on benefits, in migrants and non-migrants, in those who had been admitted to hospital for the first time when entering the study and in those who had been admitted before.

### Secondary outcomes

Unadjusted and adjusted descriptive statistics and results from multivariable regression model of secondary outcomes at the 1-year follow-up are shown in Table 3.

**Table 3.** Secondary outcomes^a^

	Total sample^b^	Personal continuity arm	Specialisation arm	Adjusted b coefficient or OR^a^ (95% confidence interval)	*p*
In-patient bed days					
*N* followed-up	6369	2313	4051		
Unadjusted mean (s.d.)	56.2 (65.7)	56.3 (63.4)	55.8 (66.2)		
Adjusted mean (s.d.)		58.3 (20.5)	53.5 (20.5)	−0.99 (−5.37 to 3.39)	0.66
Involuntary readmission					
*N* followed-up	6309	2258	4051		
Unadjusted proportion, *N* (%)	495 (7.8)	165 (7.3)	330 (8.2)		
Adjusted proportion		6.9	7.4	0.77 (0.55–1.07)	0.12
Any adverse event					
*N* followed-up	6430^c^	2313	4051		
Unadjusted proportion, *N* (%)	1127 (17.5)	425 (18.0)	701 (17.3)		
Adjusted proportion		17.4	16.9	0.93 (0.75–1.15)	0.49
SIX score at 1 year					
*N* followed-up	4260	1557	2702		
Unadjusted mean (s.d.)	3.7 (1.4)	3.6 (1.3)	3.8 (1.4)	−0.06 (−0.14 to 0.02)	0.15
Adjusted mean (s.d.)		3.6 (0.7)	3.7 (0.7)		

a The multivariable model was adjusted for the following factors: age, gender, diagnostic group, severity of illness at baseline, whether or not patient has been previously admitted, social situation (SIX score), formal status of patient at baseline and length of index hospital stay (the last factor was not adjusted for when testing in-patient bed days).

b *N* = 5 participants were not allocated to either care approach at baseline.

c *N* = 61 participants for whom data on primary outcome were not available were followed-up for adverse events.

Both in univariable and multivariable analyses, no statistically significant differences were found between the two types of care for any of the secondary outcomes, i.e., in-patient bed days in 1 year, SIX score (for social situation), involuntary readmission and occurrence of any adverse event.

## Discussion

In a very large multinational comparison of patient outcomes over a 1-year period after hospital admission, we found no statistically significant difference between patients treated by the same and those treated by different psychiatrists across in- and out-patient treatment. Re-hospitalisation rates did not significantly differ in the whole sample, in any of the five countries or in any of subgroups that were defined by socio-demographic and clinical criteria. Total number of in-patient days, involuntary admissions, adverse events and changes of the social situation did not differ significantly between the two arms either.

The strengths of this study are the multi-country comparison, the large sample size and the natural experiment design which was selected considering the limitations of previous evidence (Omer *et al*., 2015). The design enabled us to study both forms of organisation in established routine care. Further strengths are a high follow-up rate of 87% for the primary outcome, the consistent findings in various subgroups, and the consideration of different outcomes, including changes of the objective social situation of patients.

However, the design also has several weaknesses.

First, not being a randomised controlled trial, the impact of unknown confounding variables on findings cannot be ruled out.

Second, the methods for collecting follow-up data varied across countries. In Italy and the England we could rely on medical records to obtain data on re-hospitalisations, whilst in the other countries this was based on less reliable patient reports.

Third, the allocation of patients to the two different groups was decided at a system level and independently of any individual patient characteristics only in England and Italy. Other variables such as insurance arrangements and clinical decisions and patient preferences influenced them in Belgium, Germany and Poland. Yet, despite the different allocation of patients to personal continuity or specialisation in different countries, we obtained similar results in the comparison of their clinical outcomes. This may be interpreted as indicating generalisability of our results.

Fourth, we did not have symptom measures as outcomes but only proxy measures based on the use of services. Although re-hospitalisation is a widely used measure of relapse that can be reliably assessed, it can be influenced by patient preferences, clinical practice and different thresholds to admissions which may vary across countries and within countries (Dimitri *et al*., 2018).

A previous analysis of data from the index admission in this study has suggested that patients show higher initial satisfaction with in-patient treatment when the same psychiatrist is responsible for in- and out-patient treatment (Bird *et al*., 2018). This may be because patients appreciate having a psychiatrist in the hospital whom they already know and who is familiar with their history. Yet, the main criterion in the discussion about personal continuity *v*. specialisation of psychiatrists has not been increasing patient satisfaction with in-patient treatment, but achieving the best possible long-term clinical and social outcomes. As these long-term outcomes appear not to be substantially influenced by whether the same or different psychiatrists provide in- and out-patient treatment, the decision for either form of organisation must be based on other criteria. Such criteria may be patient and staff preferences, logistic considerations such as the travel time between in- and out-patient services or costs of care provision. An economic analysis is currently in progress as a specific work package of our project in order to establish whether there are significant differences in costs of care provision between the two care approaches.

Although the debate on whether the same or different psychiatrists should be responsible for in- and out-patient treatment is sometimes fierce and has driven major organisational changes and reform initiatives, the findings of this study suggest that this aspect of the organisation has less impact on long-term patient outcomes than experts may have assumed (Royal College of Psychiatrists and National Institute for Mental Health, 2005; Department of Health, 2007; Burns, 2010; Killaspy, 2012; Laugharne and Pant, 2012; Lodge, 2012; Rosen *et al*., 2013). This lack of a significant impact does not depend on specific national contexts. It applies both to countries where the allocation of patients to either form of organisation is determined by the hospital and to countries in which individual patient characteristics can influence the allocation. It also applies similarly to sub-groups of patients with different socio-demographic and clinical characteristics.

The fragmentation of care that may come with a specialised approach of in- and out-patient care might be less detrimental than sometimes assumed. Alternatively, having the same psychiatrist with responsibility for in- and out-patient care may not be sufficient to guarantee a potentially beneficial long-term continuity, particularly within the context of a multidisciplinary care team.

In any case, debates on the best form of mental health care might benefit from moving on to considering other aspects of care provision, potentially focusing more on the quality of the therapeutic relationships (Priebe and Gruyters, 1993; Priebe *et al*., 2009) rather than their mere continuity and on what types of treatment are actually provided rather than the precise organisation of the role of psychiatrists. Countries or organisations considering to switch from personal continuity to specialisation of psychiatrists for in- and out-patient treatment or vice versa might want to take the results of this study as a reason not to spend resources primarily for changing this aspect of care.

## Supplementary material

For supplementary material accompanying this paper visit http://dx.doi.org/10.1017/S2045796018000732. click here to view supplementary material

## Availability of data and materials

The authors are willing to share anonymised data upon reasonable request. Access to data needs to be subject to a formal data sharing agreement in order to ensure that any other researcher accessing the data follows appropriate standards in terms of information governance as stipulated with the national Ethics Committees. Interested researchers can contact Dr Domenico Giacco at d.giacco@qmul.ac.uk to initiate the process.
