# The same or different psychiatrists for in- and out-patient treatment? A multi-country natural experiment - ADDENDUM

**DOI:** 10.1017/S2045796019000076

**Published:** 2019-07-03

**Authors:** D. Giacco, V.J. Bird, T. Ahmad, M. Bauer, A. Lasalvia, V. Lorant, E. Miglietta, J. Moskalewicz, P. Nicaise, A. Pfennig, M. Welbel, S. Priebe

**Affiliations:** 1 Unit for Social and Community Psychiatry (World Health Organisation Collaborating Centre for Mental Health Services Development), Queen Mary University of London, London, UK; 2 Newham Centre for Mental Health, London, E13 8SP, UK; 3 Queen Mary University of London, Pragmatic Clinical Trials Unit, London, UK; 4 Centre for Primary Care and Public Health, Queen Mary University, London (Whitechapel Campus), Yvonne Carter Building, 58 Turner Street, London E1 2AB, UK; 5 Department of Psychiatry and Psychotherapy, Carl Gustav Carus University Hospital, Technische Universität Dresden, Universitätsklinikum Carl Gustav Carus, Klinik und Poliklinik für Psychiatrie und Psychotherapie, Fetscherstraße 74, 01307 Dresden, Germany; 6 UOC Psichiatria, Azienda Ospedaliera Universitaria Integrata di Verona, Piazzale Aristide Stefani, 1, 37126 Verona VR, Italy; 7 Institute of Health and Society IRSS, Université Catholique de Louvain, “Ecole de Santé Publique”, Clos Chapelle-aux-champs, 30 bte 30.15–1200 Woluwe-Saint-Lambert, Brussels, Belgium; 8 Section of Psychiatry, Department of Public Health and Community Medicine, University of Verona, Verona, Italy; 9 Institute of Psychiatry and Neurology, ul. Sobieskiego 9, 02-957 Warsaw, Poland

In the above article, a source of Financial Support was omitted. The Financial Support statement should read as follows:
