# Supplementary material for: The same or different psychiatrists for in- and out-patient treatment? A multi-country natural experiment
Source: Epidemiol Psychiatr Sci. 2018 Dec 18;29:e10. doi: 10.1017/S2045796018000732 (PMC8061192; doi:10.1017/S2045796018000732)
Supplement: Supplementary file 1 [file S2045796018000732sup001.docx]

**Supplementary material.**

**List of participating hospitals (N=57)**

**1. Clinical sites in Belgium (N=9)***

| **Study site** | **Hospital** | **Approach** |
| --- | --- | --- |
| Liège | ISoSL – Site Agora | Personal continuity or Specialisation allocated at patient level |
|  | ISoSL – Site Petit-Bourgogne | Personal continuity or Specialisation allocated at patient level |
| Charleroi | CHU de Charleroi – Site Vincent van Gogh | Personal continuity or Specialisation allocated at patient level |
| Dave (Namur) | Centre Neuropsychiatrique St Martin | Personal continuity or Specialisation allocated at patient level |
| Namur | Hôpital du Beau-Vallon | Personal continuity or Specialisation allocated at patient level |
| Ottignies | Clinique St Pierre | Personal continuity or Specialisation allocated at patient level |
| Manage | Centre Psychiatrique St Bernard | Personal continuity or Specialisation allocated at patient level |
| Kortenberg | UPC Kortenberg | Personal continuity or Specialisation allocated at patient level |
| Brussels | Clinique Fond ‘Roy and Clinique Sanatia | Personal continuity or Specialisation allocated at patient level |

1. **Clinical sites in England (N=24)**

| **Study site** | **Mental Health Trust** | **Hospitals** | **Approach** |
| --- | --- | --- | --- |
| London | East London NHS Foundation Trust | -Mile End Hospital  -Newham Centre for Mental Health | Personal continuity |
|  | North East London NHS Foundation Trust | - Goodmayes Hospital | Specialisation |
|  | Camden & Islington NHS Foundation Trust | - St Pancras Hospital  - Highgate | Specialisation |
| Essex | North Essex Partnership NHS Foundation Trust | -Colchester – The Lakes  -Chelmsford – Linden Centre | Specialisation |
|  | North Essex Partnership NHS Foundation Trust | -Harlon, Derwent Centre | Personal continuity |
| Bradford | Bradford District Care Trust | -Airedale  - Lynfield Mount | Personal continuity |
| South Staffordshire & Shropshire | South Staffordshire & Shropshire Healthcare NHS Foundation Trust | - Shropshire  -Redwoods Centre Shrewsbury  -Stafford | Specialisation |
| Cornwall | Cornwall Partnership NHS Foundation Trust | -Bodmin Hospital  - Redruth and Cambourne Community Hospital | Personal continuity |
| Dudley and Walsall | Dudley and Walsall Mental Health Partnership NHS Trust | - Walsall, Dorothy Pattison Hospital  - Dudley, Bushey Fields Hospital | Personal continuity |
| Oxford | Oxford Health NHS Foundation Trust | -Warneford hospital - Oxford)  -Littlemore hospital - Oxford  -Whiteleaf Centre - Aylesbury | Specialisation |
| Ashton-under-lyne | Pennine Care NHS Foundation Trust | Tameside hospital | Personal continuity |
| Manchester | Manchester Mental Health and Social NHS Foundation Trust | - Laureate House at Wythenshawe hospital  -Park House at North Manchester General hospital | Specialisation |

**3. Clinical sites in Germany (N=6)**

| **Study site** | **Hospital** |  |
| --- | --- | --- |
| **Dresden** | Carl Gustav Carus University hospital | Personal continuity or Specialisation allocated at patient level |
|  | Hospital Dresden Neustadt, Weißer Hirsch | Personal continuity or Specialisation allocated at patient level |
|  | St.-Marien-Krankenhaus Dresden Fachkrankenhaus für Psychiatrie, Psychotherapie und Neurologie | Personal continuity or Specialisation allocated at patient level |
| **Hamburg** | University Hospital Hamburg-Eppendorf | Personal continuity or Specialisation allocated at patient level |
| **Berlin** | St. Hedwig, Charité University Medicine | Personal continuity or Specialisation allocated at patient level |
|  | Charité-Mitte | Personal continuity or Specialisation allocated at patient level |

**4. Clinical sites in Italy (N=12)**

| **Study site** | **Hospital** | **Approach** |
| --- | --- | --- |
| Verona | Policlinico “G.B. Rossi”, Borgo Roma | Personal continuity |
|  | Ospedale Civile Maggiore, Borgo Trento | Personal continuity* |
|  | Ospedale Civile Maggiore, Borgo Trento | Specialisation* |
| San Bonifacio (VR) | Ospedale “Fracastoro” | Personal continuity |
| Bussolengo (VR) | Ospedale “Orlandi” | Specialisation |
| Isola della Scala (VR) | Ospedale di Isola della Scala | Specialisation |
| Adria (RO) | Ospedale “Santa Maria Regina degli Angeli“ | Personal continuity |
| Treviso | Ospedale “Ca’ Foncello“ | Specialisation |
| Oderzo | Ospedale Civile di Oderzo | Personal continuity |
| Montecchio Maggiore (VI) | Ospedale Montecchio Maggiore | Personal continuity |
| Vicenza | Ospedale “San Bortolo“ | Specialisation |
| Padova | Policlinico di Padova | Specialisation |
| Cittadella | Ospedale di Cittadella | Specialisation |

*Ospedale Civile Maggiore, Borgo Trento (Verona) includes two different in-patient units: 1 personal continuity and 1 specialisation

**5. Clinical sites in Poland – (N=6)**

| **Study site** | **Hospital** | **Number of wards** | **Number of beds** | **Approach** |
| --- | --- | --- | --- | --- |
| Warsaw | Psychiatric Clinic at the Institute of Psychiatry and Neurology | 2 | 72 | Personal continuity or Specialisation allocated at patient level |
|  | Nowowiejski Mental Health Hospital | 2 | 200 | Personal continuity or Specialisation allocated at patient level |
|  | Psychiatric inpatient ward in Wola Hospital | 1 | 35 | Personal continuity or Specialisation allocated at patient level |
|  | Psychiatric ward in Bielanski Hospital | 1 | 35 | Personal continuity or Specialisation allocated at patient level |
| Łomża | Psychiatric ward in Regional Hospital in Łomża | 1 | 42 | Personal continuity or Specialisation allocated at patient level |
| Grajewo | Psychiatric ward in General Hospital in Grajewo Hospital | 1 | 25 | Personal continuity or Specialisation allocated at patient level |

^*^

**Supplementary Table 1**

**Baseline socio-demographic and clinical characteristics of each country subsample**

|  | **Belgium** | **Germany** | **Italy** | **Poland** | **United Kingdom** |
| --- | --- | --- | --- | --- | --- |
|  | **(n = 1043)** | **(n = 1061)** | **(n = 1118)** | **(n = 1374)** | **(n = 2706)** |
| **Age, mean (SD)*** | 43.3 (14.3) | 41.3 (14.9) | 47.1 (14.0) | 42.3 (14.8) | 40.6 (13.5) |
| **Gender, male, N (%)** | 450 (43.1) | 558 (52.6) | 547 (48.9) | 674 (49.1) | 1582 (58.5) |
| **Partnership status, married, N (%)** | 207 (19.8) | 159 (15.0) | 330 (29.5) | 392 (28.5) | 441 (16.3) |
| **Born in the same country, yes, N (%)** | 911 (87.3) | 910 (85.8) | 980 (87.7) | 1353 (98.5) | 2144 (79.2) |
| **Education level:** |  |  |  |  |  |
| **Primary school, N (%)** | 205 (19.7) | 198 (18.7) | 102 (9.1) | 457 (33.3) | 297 (11.0) |
| **Secondary school, N (%)** | 498 (47.8) | 376 (35.5) | 481 (43.1) | 583 (42.4) | 1045 (38.6) |
| **Further education, N (%)** | 280 (26.9) | 464 (43.8) | 524 (46.9) | 329 (23.9) | 1310 (48.4) |
| **Accommodation:** |  |  |  |  |  |
| **Homeless, N (%)** | 48 (4.6) | 41 (3.9) | 7 (0.6) | 36 (2.6) | 226 (8.4) |
| **Living situation:** |  |  |  |  |  |
| **Living alone, N (%)** | 383 (36.7) | 517 (48.7) | 259 (23.2) | 287 (21.1) | 1202 (45.0) |
| **Employment:** |  |  |  |  |  |
| **Paid employment, N (%)** | 232 (22.2) | 344 (32.4) | 347 (31.1) | 451 (32.8) | 619 (22.9) |
| **Receiving benefits, yes, N (%)** | 652 (62.5) | 419 (39.5) | 337 (30.1) | 630 (45.9) | 1825 (67.4) |
| **Diagnosis at admission:** |  |  |  |  |  |
| **Psychotic disorders, N (%)** | 328 (31.5) | 347 (32.7) | 425 (38.0) | 737 (53.6) | 1154 (42.6) |
| **Mood disorders, N (%)** | 606 (58.1) | 675 (63.6) | 541 (48.4) | 407 (29.6) | 1369 (50.6) |
| **Anxiety, dissociative, stress-related, and somatoform disorders, N (%)** | 211 (20.2) | 278 (26.2) | 157 (14.0) | 264 (19.2) | 427 (15.8) |
| **First admission, yes, N (%)** | 366 (35.1) | 355 (33.5) | 342 (30.6) | 460 (33.5) | 912 (33.7) |
| **Voluntary admission, yes, N (%)** | 869 (83.3) | 987 (93.0) | 1023 (91.5) | 1238 (90.1) | 1550 (57.3) |
| **Personal continuity, yes, N (%)** | 483 (46.3) | 216 (20.4) | 368 (32.9) | 405 (29.5) | 1094 (40.4) |
| **Specialisation, yes , N (%)** | 555 (53.2) | 842 (79.4) | 750 (67.1) | 967 (70.3) | 1612 (59.6) |
| **Clinical Global Impression score, mean (SD)** | 3.2(0.9) | 4.8(0.9) | 4.6(0.8) | 4.2(1.0) | 4.4(1.4) |
| **Index length of stay, mean (SD)** | 55.1 (62.4) | 37.0 (29.2) | 17.9 (16.6) | 33.4 (28.1) | 46.2 (63.0) |
| **SIX score at baseline, mean (SD)** | 3.6 (1.4) | 3.7 (1.4) | 3.9 (1.3) | 4.1 (1.3) | 3.3 (1.5) |

***SD = standard deviation**

**Supplementary Table 2**

**Baseline socio-demographic and clinical characteristics by diagnostic groups**

|  | | |  |  | |  |
| --- | --- | --- | --- | --- | --- | --- |
|  | **F2** | **F3** | | | **F4** | |
|  | **(n = 2991)** | **(n= 3598)** | | | **(n = 1337)** | |
| **Age, mean (SD)*** | 40.4 (13.6) | 44.6 (14.5) | | | 40.1 (14.5) | |
| **Gender, male, N (%)** | 1856 (62.2) | 1641 (45.6) | | | 632 (47.3) | |
| **Partnership status, married, N (%)** | 507 (16.9) | 1106 (30.7) | | | 371 (27.7) | |
| **Born in the same country, yes, N (%)** | 2528 (84.5) | 3108 (86.4) | | | 1999 (89.7) | |
| **Education level:** |  |  | | |  | |
| **Primary school, N (%)** | 596 (19.9) | 533 (14.8) | | | 233 (17.4) | |
| **Secondary school, N (%)** | 1260 (42.1) | 1432 (39.8) | | | 551 (41.2) | |
| **Further education, N (%)** | 1061 (35.5) | 1565 (43.5) | | | 526 (39.3) | |
| **Accommodation:** |  |  | | |  | |
| **Homeless, N (%)** | 175 (5.8) | 152 (4.2) | | | 58 (4.3) | |
| **Living situation:** |  |  | | |  | |
| **Living alone, N (%)** | 1167 (39.8) | 1286 (35.7) | | | 440 (32.9) | |
| **Employment:** |  |  | | |  | |
| **Paid employment, N (%)** | 554 (19.5) | 1171 (32.5) | | | 462 (34.5) | |
| **Receiving benefits, yes, N (%)** | 1895 (63.4) | 1705 (47.4) | | | 592 (44.3) | |
| **First admission, yes, N (%)** | 686 (22.9) | 1316 (36.6) | | | 651 (48.7) | |
| **Voluntary admission, yes, N (%)** | 2069 (69.2) | 2926 (81.3) | | | 1192 (89.1) | |
| **Personal continuity, yes, N (%)** | 1128 (37.7) | 1218 (33.8) | | | 458 (34.3) | |
| **Specialisation, yes, N(%)** | 1857 (62.1) | 2375 (66.0) | | | 878 (65.7) | |
| **Clinical Global Impression score, mean (SD)** | 4.5 (1.1) | 4.29 (1.2) | | | 3.93 (1.2) | |
| **Index length of stay, mean (SD)** | 47.0 (59.9) | 36.7 (42.7) | | | 32.9 (42.2) | |
| **SIX score at baseline, mean (SD)** | 3.3 (1.4) | 3.9 (1.4) | | | 3.9 (1.4) | |

***SD = standard deviation**

**Supplementary Table 3**

**Baseline socio-demographic and clinical characteristics by gender**

|  | **Female** | **Male** |
| --- | --- | --- |
|  | **(n = 3475)** | **(n= 3811)** |
| **Age, mean (SD)** | 44.2 (14.5) | 40.7 (13.9) |
| **Partnership status, married, N (%)** | 913 (26.4) | 613 (16.1) |
| **Born in the same country, yes, N (%)** | 446 (12.9) | 534 (14.0) |
| **Education level:** |  |  |
| **Primary school, N (%)** | 572 (16.6) | 684 (18.0) |
| **Secondary school, N (%)** | 1381 (40.0) | 1596 (42.1) |
| **Further education, N (%)** | 1452 (42.0) | 1448 (38.2) |
| **Accommodation:** |  |  |
| **Homeless, N (%)** | 93 (2.7) | 264 (6.9) |
| **Living situation:** |  |  |
| **Living alone, N (%)** | 1120 (32.6) | 1521 (40.8) |
| **Employment:** |  |  |
| **Paid employment, N (%)** | 959 (27.7) | 1031 (27.2) |
| **Receiving benefits, yes, N (%)** | 1767 (51.4) | 2087 (55.2) |
| **Diagnosis at admission:** |  |  |
| **Psychotic disorders, N (%)** | 1130 (32.5) | 1856 (48.7) |
| **Mood disorders, N (%)** | 1945 (56.0) | 1641 (43.1) |
| **Anxiety, dissociative, stress-related, and somatoform disorders, N (%)** | 703 (20.2) | 632 (16.6) |
| **First admission, yes, N (%)** | 1174 (34.1) | 1255 (33.1) |
| **Voluntary admission, yes, N (%)** | 2819 (81.3) | 2839 (74.7) |
| **Personal continuity, yes, N (%)** | 1189 (34.3) | 1368 (35.9) |
| **Specialisation, yes, N(%)** | 2280 (65.7) | 2439 (64.1) |
| **Clinical Global Impression score, mean (SD)** | 4.3 (1.2) | 4.4 (1.2) |
| **Index length of stay, mean (SD)** | 39.0 (48.2) | 39.8 (51.1) |
| **SIX score at baseline, mean (SD)** | 3.8 (1.3) | 3.5 (1.5) |

***SD = standard deviation**

**Supplementary Table 4**

**Baseline socio-demographic and clinical characteristics by age group (<40 years; => 40 years)**

|  | **<=40 years** | **> 40 years** |
| --- | --- | --- |
|  | **(n = 3364)** | **(n=3891)** |
| **Gender, male, N (%)** | 1966 (58.5) | 1833 (46.9) |
| **Partnership status, married, N (%)** | 406 (12.1) | 1120 (28.6) |
| **Born in the same country, yes, N (%)** | 532 (15.9) | 452 (11.5) |
| **Education level:** |  |  |
| **Primary school, N (%)** | 518 (15.5) | 738 (18.9) |
| **Secondary school, N (%)** | 1353 (40.4) | 1623 (60.6) |
| **Further education, N (%)** | 1434 (42.8) | 1466 (37.7) |
| **Accommodation:** |  |  |
| **Homeless, N (%)** | 224 (6.7) | 133 (3.4) |
| **Living situation:** |  |  |
| **Living alone, N (%)** | 1033 (31.4) | 1610 (41.6) |
| **Employment:** |  |  |
| **Paid employment, N (%)** | 933 (27.8) | 1053 (26.9) |
| **Receiving benefits, yes, N (%)** | 1664 (49.8) | 2192 (56.5) |
| **Diagnosis at admission:** |  |  |
| **Psychotic disorders, N (%)** | 1620 (48.2) | 1365 (34.8) |
| **Mood disorders, N (%)** | 1381 (41.0) | 2208 (56.3) |
| **Anxiety, dissociative, stress-related, and somatoform disorders, N (%)** | 680 (20.2) | 653 (16.6) |
| **First admission, yes, N (%)** | 1306 (39.1) | 1124 (28.9) |
| **Voluntary admission, yes, N (%)** | 2551 (76.1) | 3099 (79.2) |
| **Personal continuity, yes, N (%)** | 1172 (34.9) | 1391 (35.5) |
| **Specialisation, yes, N(%)** | 2185 (65.1) | 2527 (64.5) |
| **Clinical Global Impression score, mean (SD)** | 4.3 (1.2) | 3.7 (4.3) |
| **Index length of stay, mean (SD)** | 40.6 (54.3) | 38.3 (45.5) |
| **SIX score at baseline, mean (SD)** | 3.8 (1.5) | 3.6 (1.4) |

***SD = standard deviation**

**Supplementary Table 5**

**Baseline socio-demographic and clinical characteristics by socio-economic status (being on benefits)**

|  | **On benefits** | **Not on benefits** |
| --- | --- | --- |
|  | **(n = 3863)** | **(n=3373)** |
| **Age, mean (SD)** | 43.1 (13.4) | 41.6 (15.2) |
| **Gender, male, N (%)** | 2087 (54.1) | 1696 (50.4) |
| **Partnership status, married, N (%)** | 625 (16.2) | 890 (26.4) |
| **Born in the same country, yes, N (%)** | 486 (12.6) | 493 (14.7) |
| **Education level:** |  |  |
| **Primary school, N (%)** | 818 (21.3) | 434 (12.9) |
| **Secondary school, N (%)** | 1649 (42.9) | 1311 (39.0) |
| **Further education, N (%)** | 1299 (33.8) | 1584 (47.1) |
| **Accommodation:** |  |  |
| **Homeless, N (%)** | 204 (5.3) | 152 (4.5) |
| **Living situation:** |  |  |
| **Living alone, N (%)** | 1632 (42.9) | 992 (29.9) |
| **Diagnosis at admission:** |  |  |
| **Psychotic disorders, N (%)** | 1895 (41.1) | 1071 (31.7) |
| **Mood disorders, N (%)** | 1705 (44.1) | 1858 (55.1) |
| **Anxiety, dissociative, stress-related, and somatoform disorders, N (%)** | 592 (15.3) | 735 (21.8) |
| **First admission, yes, N (%)** | 888 (23.2) | 1525 (25.4) |
| **Voluntary admission, yes, N (%)** | 2841 (73.7) | 2776 (82.5) |
| **Personal continuity, yes, N (%)** | 1484 (38.5) | 1058 (31.4) |
| **Specialisation, yes, N(%)** | 2370 (61.5) | 2314 (68.6) |
| **Clinical Global Impression score, mean (SD)** | 3.6 (4.3) | 4.3 (1.1) |
| **Index length of stay, mean (SD)** | 45.2 (57.8) | 32.7 (37.7) |
| **SIX score at baseline, mean (SD)** | 3.2 (1.3) | 4.2 (1.4) |

***SD = standard deviation**

**Supplementary Table 6**

**Baseline socio-demographic and clinical characteristics by migrant status (not being born in the country in which the participant is hospitalised)**

|  | **Migrant** | **Non-migrant** |
| --- | --- | --- |
|  | **(n =985)** | **(n=6298)** |
| **Age, mean (SD)** | 39.4 (12.8) | 42.9 (14.4) |
| **Gender, male, N (%)** | 534 (54.5) | 3270 (52.0) |
| **Partnership status, married, N (%)** | 220 (22.4) | 1306 (20.8) |
| **Education level:** |  |  |
| **Primary school, N (%)** | 122 (12.4) | 1134 (18.1) |
| **Secondary school, N (%)** | 351 (35.8) | 2626 (41.9) |
| **Further education, N (%)** | 484 (49.4) | 2417 (38.6) |
| **Accommodation:** |  |  |
| **Homeless, N (%)** | 87 (8.8) | 270 (4.3) |
| **Living situation:** |  |  |
| **Living alone, N (%)** | 392 (40.9) | 2252 (36.3) |
| **Employment:** |  |  |
| **Paid employment, N (%)** | 247 (25.1) | 1739 (27.7) |
| **Receiving benefits, yes, N (%)** | 486 (49.6) | 3368 (54.0) |
| **Diagnosis at admission:** |  |  |
| **Psychotic disorders, N (%)** | 455 (46.2) | 2528 (40.1) |
| **Mood disorders, N (%)** | 482 (48.9) | 3108 (49.3) |
| **Anxiety, dissociative, stress-related, and somatoform disorders, N (%)** | 134 (13.6) | 1199 (19.0) |
| **First admission, yes, N (%)** | 374 (38.3) | 2058 (32.9) |
| **Voluntary admission, yes, N (%)** | 637 (64.9) | 5017 (79.9) |
| **Personal continuity, yes, N (%)** | 389 (39.5) | 2171 (34.5) |
| **Specialisation, yes, N(%)** | 595 (60.5) | 4118 (65.5) |
| **Clinical Global Impression score, mean (SD)** | 4.4 (1.2) | 4.3 (1.2) |
| **Index length of stay, mean (SD)** | 43.0 (55.7) | 38.8 (48.6) |
| **SIX score at baseline, mean (SD)** | 3.4 (1.5) | 3.7 (1.4) |

***SD = standard deviation**

**Supplementary Table 7**

**Baseline socio-demographic and clinical characteristics of people at first admission and of those with a history of previous admissions (repeated admission)**

|  | **First admission** | **Repeated admission** |
| --- | --- | --- |
|  | **(n =2435)** | **(n=4814)** |
| **Age, mean (SD)** | 39.9 (14.7) | 43.7 (13.9) |
| **Gender, male, N (%)** | 1255 (51.7) | 2536 (52.8) |
| **Partnership status, married, N (%)** | 596 (24.6) | 924 (19.2) |
| **Born in the same country, yes, N (%)** | 374 (15.4) | 603 (12.6) |
| **Education level:** |  |  |
| **Primary school, N (%)** | 347 (14.3) | 908 (18.9) |
| **Secondary school, N (%)** | 992 (40.8) | 1971 (41.1) |
| **Further education, N (%)** | 1051 (43.3) | 1844 (38.4) |
| **Accommodation:** |  |  |
| **Homeless, N (%)** | 124 (5.1) | 233 (4.8) |
| **Living situation:** |  |  |
| **Living alone, N (%)** | 752 (31.3) | 1872 (39.6) |
| **Employment:** |  |  |
| **Paid employment, N (%)** | 961 (39.5) | 1024 (21.4) |
| **Receiving benefits, yes, N (%)** | 888 (36.8) | 2939 (61.5) |
| **Diagnosis at admission:** |  |  |
| **Psychotic disorders, N (%)** | 686 (28.2) | 2286 (47.5) |
| **Mood disorders, N (%)** | 1316 (54.1) | 2254 (46.8) |
| **Anxiety, dissociative, stress-related, and somatoform disorders, N (%)** | 651 (26.7) | 674 (14.0) |
| **Voluntary admission, yes, N (%)** | 1948 (80.4) | 3677 (76.5) |
| **Personal continuity, yes, N (%)** | 824 (33.9) | 1728 (35.9) |
| **Specialisation, yes, N(%)** | 1608 (66.1) | 3079 (64.1) |
| **Clinical Global Impression score, mean (SD)** | 4.2 (1.2) | 4.4 (1.2) |
| **Index length of stay, mean (SD)** | 32.3 (40.6) | 42.7 (53.2) |
| **SIX score at baseline, mean (SD)** | 4.1 (1.4) | 3.5 (1.4) |

***SD = standard deviation**
